# The influence of academic pressure on adolescents’ problem behavior: Chain mediating effects of self-control, parent–child conflict, and subjective well-being

**DOI:** 10.3389/fpsyg.2022.954330

**Published:** 2022-09-21

**Authors:** Mao-min Jiang, Kai Gao, Zheng-yu Wu, Pei-pei Guo

**Affiliations:** ^1^School of Public Affairs, Xiamen University, Xiamen, China; ^2^School of Management, Shanghai University of Engineering Sciences, Shanghai, China

**Keywords:** teenagers, academic pressure, problem behavior, self-control, parent–child conflict, subjective well-being, chain mediation

## Abstract

As a negative social issue, teenagers’ problem behavior not only affects individuals’ physical and mental health and social function development but is also not conducive to social harmony and stability. This study mainly discusses the influence of academic pressure on adolescents’ problem behavior, and the potential relationship between these and academic pressure, examining issues such as self-control, parent–child conflict, and subjective well-being. The data were collected from the fifth wave of the China Family Panel Studies (2017–2018). The data of 2,465 teenagers aged 10–15 were analyzed by LISREL8.8 software. The results show that academic pressure positively affects adolescents’ deviant behavior. The mediation model finds that parent–child conflict and self-control play a direct mediating role between academic pressure and adolescents’ behavioral problems. Parent–child conflict, self-control, and subjective well-being have important chain mediation effects between academic pressure and adolescents’ problem behavior. Therefore, in order to reduce the risk of such problems, it is necessary to further strengthen individuals’ ability to maintain self-control, promote or cultivate adolescents’ character strengths, create a harmonious family atmosphere, reduce the probability of parent–child conflict, and increase the subjective well-being of teenagers.

## Introduction

Adolescents’ problem behavior refers to behavior that deviates from the normal standard of society expected while adolescents are growing up (Markova and Nikitskaya, 2014). As a negative social behavior, it is usually used to measure adolescents’ physical and mental health and social function development (Kenneth et al., 2003). The specific manifestations of adolescents’ problem behavior include an inappropriate learning attitude, poor interpersonal relationships, and bad living habits (Kremer et al., 2016). Relevant research shows that adolescents’ problem behavior is persistent, which can significantly affect adult drinking, violence, and even committing crimes (Pol et al., 2012; Evans et al., 2020). This not only affects adolescents’ behavioral health, but also is not conducive to the harmonious and stable development of society. It has been found that adolescents’ problem behavior is mainly influenced by individuals, families, schools, and society, and the disharmonious parent–child relationship, school violence, and social order are all important factors that lead to it (Reaves et al., 2018). This study seeks to verify the formation mechanism and path of influence of adolescent problem behaviors from the four levels of individual, family, school, and society, starting from the external environment and internal performance, through to structural equation modeling, in order to find ways reduce adolescent problem behavior and promote the development of positive social functions.

Given the background of China’s nine-year compulsory education system, teenagers are not able to live without learning during their long growth stage. Academic pressure may have an important impact on teenagers’ physical and mental health, family relations, and happiness, which has been confirmed by many studies (Sun et al., 2011; Zhang et al., 2016). Academic pressure refers to the tension, discomfort, and other emotions caused by the pressure from school, family, and society in the learning process (Luo et al., 2020). Studies have shown that teachers and parents have higher learning expectations of teenagers with good academic performance, resulting in greater academic pressure. When academic performance does not match expectations, this can create negative emotions, which will lead to deviant behaviors (Ma et al., 2018; Çelik, 2019). Teenagers with poor academic performance are vulnerable to peer pressure in the campus environment, and they are prone to feelings of inferiority, anxiety, and fear in their studies. At the same time, their academic failures also make them vulnerable to peer investigation and rejection. This leads to rebellious psychological issues, showing problem behavior such as hyperactivity and aggression, and even crimes (McEvoy and Welker, 2000). Therefore, it is particularly important to study the path of influence of academic pressure on adolescents’ problem behavior.

Parent–child relationships, teacher–student relationships, and peer relationships are the most important social relationships of teenagers. They play an important role in the development of their social functions (Buist, 2004). The parent–child relationship is a type of interpersonal relationship that unites a natural relationship and a social relationship. It is bound by blood relationship and has the particularity of stability, permanence, intimacy, and an appropriate relationship. It has an extremely important influence on the physical and mental development of teenagers (Kerri et al., 2009). It has been found that parents’ psychological aggression and corporal punishment can easily lead to more deviant behaviors, such as aggression, violation of discipline, and anxiety. Parent–child conflict has a long-term and persistent negative impact on adolescents’ behavioral problems (Bradford et al., 2007). Some studies have found that parent–child conflict has a moderating effect on academic pressure and adolescent behavioral deviation (Kuang, 2011). In addition, a number of studies have also found that academic pressure may affect adolescents’ problem behavior through parent–child conflict. Academic pressure includes the pressure placed on adolescents by parents. When academic performance fails to satisfy parents, it will affect family relations, resulting in parent–child conflict, which may lead to an increase in adolescents’ problem behavior (Huan et al., 2008). The way parent–child conflict affects the relationship between academic pressure and adolescents’ problem behavior needs further verification.

Self-control is one of the most important factors that affect an individual’s internal development. According to changes in the external environment, a person can adjust their thoughts, emotions, and behaviors in time to achieve established goals (Casey and Caudle, 2013). Relevant research proves that teenagers with high self-control have greater happiness, excellent academic performance, good interpersonal relationships, less mental illness and problem behavior, and, at the same time, they can better regulate negative personal emotions (Pokhrel et al., 2017). A study found that academic pressure can regulate adolescents’ problem behavior through individual self-control. Strong self-control ability can also relieve academic pressure and reduce negative emotions (Xu et al., 2018). In addition, the parent–child relationship can also influence adolescents’ self-control and then their problem behavior. For example, self-control theory points out that a negative parent–child relationship will reduce the development of adolescents’ self-control ability. However, the lack of self-control ability can easily lead to individual bad behavior (Bunch et al., 2017). A general theory of crime also shows that parent–child conflict is an important factor restricting the development of adolescents’ self-control ability, and adolescents with low self-control ability are at high risk of violating discipline or committing crimes (Hirschi and Gottfffredson, 1993). Therefore, the direct and indirect effects of academic pressure, parent–child relationship, and self-control on adolescents’ problem behavior still need to be further explored.

Subjective well-being is an indicator of an individual’s life state ascertained by examining certain standards, mainly emotional state and life satisfaction (Diener and Fujita, 1995). Subjective well-being, as a psychological characteristic of teenagers, can effectively measure their social adaptation state, and it is also an important index to evaluate their psychological adaptability (Eryılmaz, 2011). Studies have found that subjective well-being can not only effectively predict adolescents’ mental health, but also predict their tendency to display problem behavior (Rask et al., 2002). Teenagers with low subjective well-being often show anxiety, loneliness, depression, etc. They often deal with social relations with a negative attitude, and their social adaptation is low, which is more likely to lead to problem behavior (Arslan and Renshaw, 2017). Subjective well-being can also affect teenagers’ problem behavior through academic pressure, the parent–child relationship, and self-control. The greater the academic pressure, the worse the parent–child relationship, and the lower the self-control ability; children’s subjective well-being is often low, thus increasing the risk of problem behavior (Qutaiba et al., 2012; Bücker et al., 2018). Therefore, it is particularly important to explore the influence mechanism of subjective well-being on adolescents’ problem behavior.

**Figure 1:**
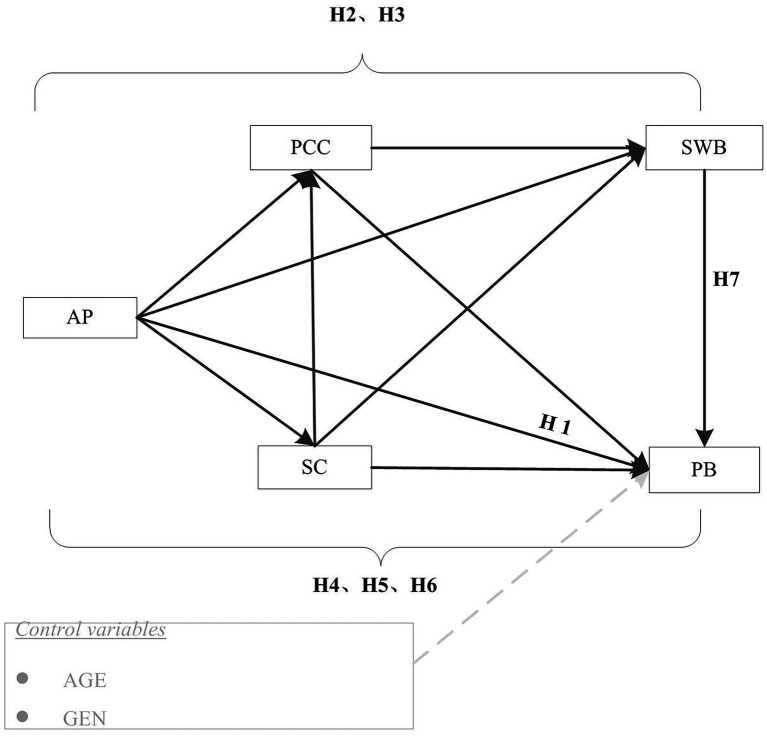
Hypothesized model of the research framework.

What is the relationship between group academic pressure and the behavioral problems of teenagers? Do self-control, parent–child conflict, and subjective well-being have mediating effects between academic pressure and adolescents’ problem behavior? Based on the existing theoretical basis and literature, the research hypotheses are shown in Figure 1: (H1) academic pressure is positively correlated with adolescents’ problem behavior; (H2) parent–child conflict has a mediating effect on the relationship between academic pressure and adolescents’ problem behavior; (H3) parent–child conflict and subjective well-being have chain mediation between academic pressure and adolescents’ problem behavior; (H4) self-control has a potential mediating effect in the relationship between academic pressure and adolescents’ problem behavior; (H5) self-control and subjective well-being have chain mediation between academic pressure and adolescents’ problem behavior; (H6) parent–child conflict, self-control, and subjective well-being have complex chain mediation between academic pressure and adolescents’ problem behavior; and (H7) subjective well-being plays an intermediary role in academic pressure and adolescents’ problem behavior.

## Materials and methods

### Participants

The data were collected from the China Family Panel Studies (CFPS) in 2018. This is a nationwide, large-scale, multi-disciplinary social follow-up survey project, focusing on the economic activities, educational achievements, family relations and family dynamics, health, and other topics of Chinese residents. A baseline survey was officially carried out in 25 provinces, cities, and autonomous regions in China in which 14,960 households and 42,590 individuals were interviewed. The permanent tracking objects of the CFPS survey are visited every 2 years. First, a total of 37,354 people participated in the fifth wave survey. According to the characteristics of the research subjects, 34,747 people were selected. Then, 142 non-school and blank questionnaires were excluded. The final sample comprised 2,465 people. The CFPS project has been approved by Peking University Biomedical Ethics Committee (IRB00001052-14010). All the participants signed an informed consent form.

### Measures

#### Academic pressure

Academic pressure was mainly measured by the degree of stress experienced by the participants during their own studies. This was measured by asking the following question: “How much do you think your academic pressure is?” The answer was based on five grades (no pressure to great pressure, with a score of 1–5). The higher the score, the greater the academic pressure on teenagers.

#### Parent–child conflict

This indicator was measured by asking the respondents how many times they had quarreled with their parents in the last month. The answers were defined as five grades (1 = 0 times, 2 = 1–2 times; 3 = 3–4 times, 4 = 5–6 times, 5 = 7 times and above).

#### Self-control

The CFPS has investigated the self-control ability of teenagers aged 10 to 15 since 2012. This scale mainly consists of 12 items, which are used to evaluate the self-control state of daily behavior. It includes the following 12 statements: (1) I am always well prepared, (2) I pay attention to details, (3) I like to be organized, (4) I will do things according to my own schedule, (5) I am very careful in my study, (6) I always put things at random, (7) I always mess things up, (8) I always forget to restore things, (9) I do things carefully and thoroughly, (10) I do my homework first and then play, (11) I’ll start my homework assignment right after, and (12) I’ll clean up when things get messy. Questions six (I always put things at random), seven (I always mess things up), and eight (I always forget to restore things) are reverse questions. Their answers were scored in reverse before the analysis in this study. Each item was rated from 1 to 5, where 1 = strongly disagree, 2 = disagree, 3 = neither agree nor disagree, 4 = agree, and 5 = quite agree. The higher the score, the stronger the self-control ability.

#### Subjective well-being

Subjective well-being was mainly measured by asking whether participants thought they were happy or not. It was measured by asking the participants, “Do you think you are happy?” Their responses included 11 grades (0–10). The higher the score, the higher the happiness index.

#### Problem behavior

In the CFPS 2018 Personal Questionnaire, information was collected about adolescent respondents aged 10 to 15 for the first time, including internalizing and externalizing problem behavior. In 2018, the CFPS adopted a more concise version (Myers et al., 1994) from the Early Childhood Liberal Study in the United States, which contains 14 questions, including eight internalized questions and six externalized questions. Internalized questions related to the following: angry, exam, loneliness, sadness, performance, worry about homework, playmates, and shame; externalization included: quarreling, paying attention, being distracted, finishing homework, being talkative, and fighting. Each entry was rated from 1 to 5, where 1 = totally non-conforming, 2 = non-conforming, 3 = fair, 4 = relatively consistent, and 5 = completely consistent. The higher the score, the greater the probability of adolescent deviant behavior.

### Data analysis

In this study, SPSS22 was used to analyze the correlation between variables and the frequency, mean, and standard deviation of each index. The Cronbach’s alpha coefficient was obtained by factor analysis to evaluate the internal consistency of the scale. LISREL8.80 software was used to construct the chain structure equation, and the intermediary effect was verified. The moderating variables were parent–child conflict, self-control, and subjective happiness, and the independent variable was academic pressure. The dependent variable was problem behavior (Figure 1). To explore the fitting effect of the structural equation model, the values of the comparative fitting index (CFI), non-normalized fitting index (NNFI), incremental fitting index (IFI), and modified goodness-of-fit index (AGFI) were limited to be higher than 0.90 (Bentler, 1990; Hu and Bentler, 1999). The approximate root mean error (RMSEA) value was <0.05 (Steiger, 1990; Browne and Cudeck, 1992). The critical value (CN) of Hoelter was greater than 200 (Bollen, 1986).

## Results

### Descriptive data

Table 1 shows the main demographic characteristics of the teenagers investigated, in which the average age is 12.40 (*SD* = 1.66) years old. There is little difference between the number of boys and girls in the survey. Those who have never quarreled with their parents in the past month account for 73.24% of the total number, and only 3.76% of teenagers have quarreled with their parents five times or more in the past month. The average score of adolescents’ academic pressure is 2.89 (*SD* = 1.14), the average score of self-control is 42.28 (*SD* = 6.76), the average score of subjective well-being is 8.15 (*SD* = 2.09), and the average score of problem behavior is 31.83 (*SD* = 8.16).

**Table 1:** Descriptive statistics variables of the sample (*n* = 2,465).

Variable	*n* ^#^	%	Mean	SD
*Control variable*				
Age [10–15]			12.40	1.66
Sex				
Male	1,305	52.94		
Female	1,160	47.06		
*Independent variables*				
Academic pressure [1–5]			2.89	1.14
*Mediating variables*				
Parent–child conflict				
Number of quarrels with parents				
never	1,697	73.24		
1–2 times	415	17.91		
3–4 times	118	5.09		
5–6 times	44	1.90		
≥7 times	43	1.86		
Self-control [12–60]			42.28	6.76
Subjective well-being [1–10]			8.15	2.09
*Dependent variable*				
Problem behavior [6–30]			31.83	8.16

*Note*: Total number < *n* = 2,465 due to missing data.

[], The range of a single item.

### Mediation analyses

LISREL 8.8 software was used to perform confirmative factor analysis of the measurement indicators. The analysis results are shown in the following table. The RMSEA values of self-control and behavioral deviation are all less than 0.08, the values of NNFI, CFI, IFI, and AGFI are all greater than 0.9, the Cronbach’s alpha values are all greater than 0.7, and the reliability and validity of the scale are within the acceptable range (Table 2).

**Table 2:** Scale reliability and validity tests.

Variables	RMSEA	NNFI	CFI	IFI	AGFI	CN	Cronbach’s alpha
Self-control	0.063	0.93	0.95	0.95	0.97	330.83	0.874
Problem behavior	0.069	0.90	0.93	0.93	0.96	271.42	0.854

**Figure 2:**
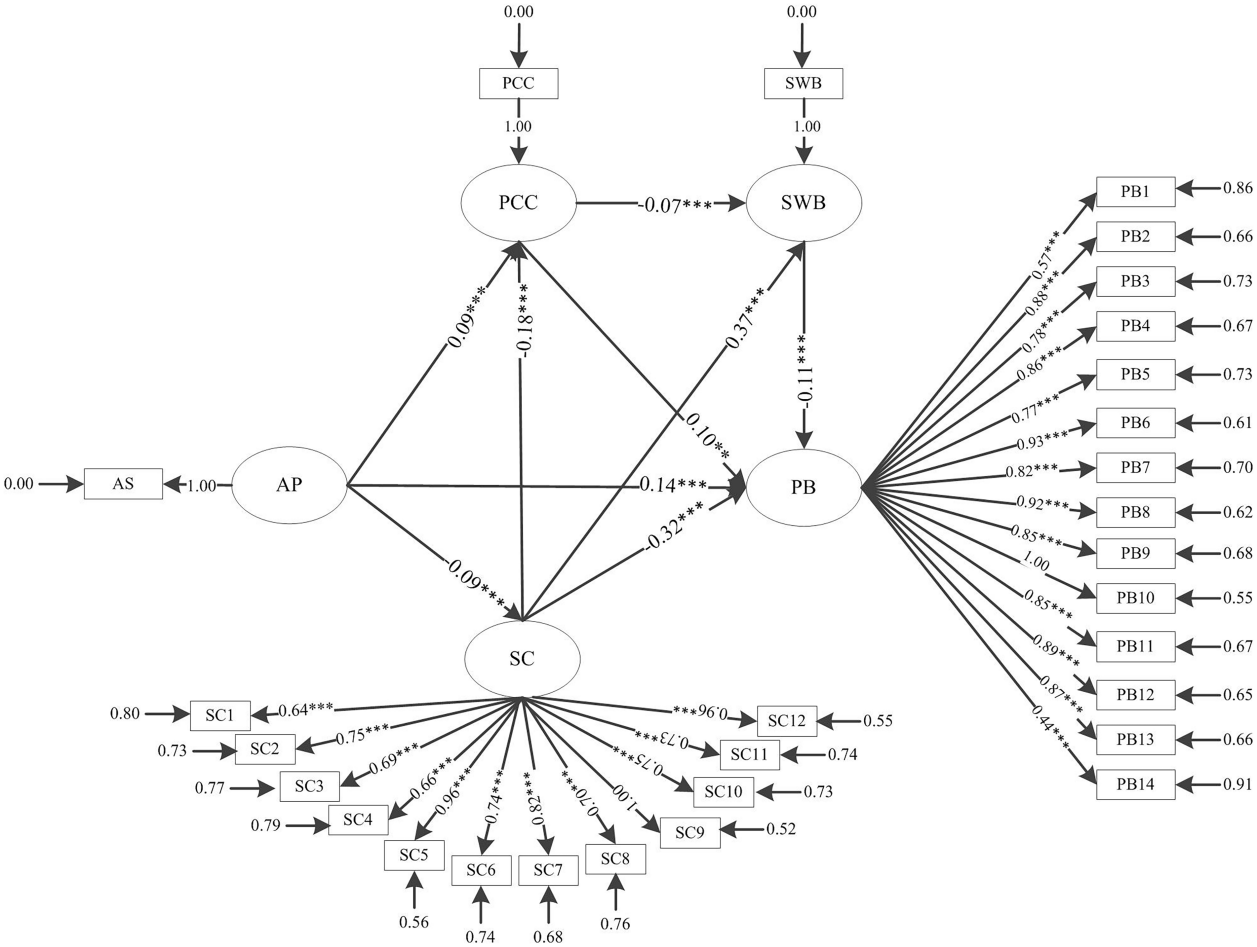
Structural equation modeling results. *Note*: AP, academic pressure (the influence of academic pressure on adolescent problem behavior); SC, self-control; PCC, parent–child conflict; SWB, subjective well-being; PB, problem behavior; **p < 0.01, ***p < 0.001.

According to the value of *t* (*t* < 1.96), in order to obtain the final model (Figure 2), the insignificant path was eliminated by using the structural equation model. The model has a good fitting degree, RMSEA = 0.043, NNFI = 0.90, CFI = 0.93, IFI = 0.93, AGFI = 0.98, CN = 470.35 (Table 3). The results show that the total effect was 0.082 (95% CI 0.64–0.102), the total indirect effect was 0.008 (95% CI 0.03–0.014), the direct effect was 0.074 (95% CI 0.56–0.092), all *p* < 0.01, and the model was some intermediaries (Table 4). In addition, academic pressure, parent–child conflict, and adolescent deviation have significant positive effects, while self-control and subjective well-being have significant negative effects on adolescent deviation. Academic pressure is negatively correlated with self-control, positively correlated with parent–child conflict, and has no direct impact on subjective well-being. Self-control, parent–child relationship, and subjective well-being play a significant chain-mediated role in academic pressure and adolescent deviant behavior.

**Table 3:** Measures of goodness-of-fit for academic pressure and problem behavior model of the adolescents.

Model	Chi-square	*df*	RMSEA	NNFI	CFI	IFI	AGFI	CN
Initial model	1688.49	418	0.043	0.90	0.93	0.93	0.98	469.39
Delete AGE → PCC	1690.77	419	0.043	0.90	0.93	0.93	0.98	469.79
Delete AP → SWB	1692.92	420	0.043	0.90	0.93	0.93	0.98	470.79
Delete AGE → PB	1693.77	421	0.043	0.90	0.93	0.93	0.98	471.03
Delete GEN → PCC	1694.02	422	0.043	0.90	0.93	0.93	0.98	471.99
Delete GEN → SWB	1707.41	423	0.043	0.90	0.93	0.93	0.98	469.32
Delete AGE → SWB #	1707.41	424	0.043	0.90	0.93	0.93	0.98	470.35

IA, internet addiction; PB, problem behavior; GEN, gender; SC, self-control; DP18, depression in 2017–2018.

# Goodness-of-fit of the final model.

**Table 4:** Analysis and comparison of specific direct and indirect effects.

	Point estimate	Product of coefficients			BOOTSTRAP1000 TIMES 95% CI			
					Bias corrected		Percentile	
	Estimate	S.E.	Est./S.E.	*p*-Value	Lower	Upper	Lower	Upper
Total effect	0.082	0.010	8.207	***	0.063	0.099	0.064	0.102
Total indirect effect	0.008	0.003	2.986	**	0.003	0.015	0.003	0.014
Direct effect	0.074	0.009	7.906	***	0.057	0.093	0.056	0.092

Table 5 shows the path of influence between academic pressure and adolescents’ problem behavior. The study found that academic pressure has a significant influence on adolescents’ problem behavior, with a total effect of 0.19 (*p* < 0.001) and a direct effect of 0.14 (*p* < 0.001). Hypothesis 1 is supported by the data. Academic pressure has a positive impact on parent–child conflict (*β* = 0.09, *p* < 0.001), and parent–child conflict also has a direct impact on adolescents’ problem behavior (*β* = 0.10, *p* < 0.01). It has a significant negative effect on subjective well-being (*β* = −0.07, *p* < 0.001), and subjective well-being can directly affect adolescents’ problem behavior (*β* = −0.11, *p* < 0.001). Therefore, academic pressure can positively affect adolescents’ problem behavior through parent–child conflict, and it can also affect adolescents’ problem behavioral through parent–child conflict and subjective well-being. Hypotheses 2, 3, and 7 are supported to some extent. In addition, academic pressure negatively affects self-control (*β* = −0.09, *p* < 0.001), while self-control directly affects adolescents’ parent–child conflict, subjective well-being, and problem behavior, with direct effects of −0.18, 0.37, and −0.32, respectively (*p* < 0.001). Therefore, academic pressure can affect adolescents’ problem behavior through self-control. The deviant behavior of teenagers can be influenced through self-control and parent–child conflict, but also through self-control, parent–child conflict, and subjective well-being. Therefore, hypotheses 4, 5, and 6 are supported to some extent.

**Table 5:** Direct and indirect effects of academic pressure and problem behavior in adolescents.

Variables	Problem behavior		
	Direct effect	Indirect effect	Total effect
*Independent variables*			
Academic pressure	0.14	0.05	0.19
*Mediation variables*			
Parent–child conflict	0.10	0.01	0.11
Self-control	-0.32	−0.06	−0.38
Subjective well-being	−0.11	-	−0.11

## Discussion

From the four levels of individual, family, school, and society, this study took the external environment and internal performance as the starting point to explore the formation mechanism and path of influence of adolescent problem behavior, so as to improve the references for reducing such behavior and promoting the development of positive social functions. Using a structural equation model, this study verified the path of influence of academic pressure on adolescent problem behavior. It has shown that academic pressure positively influenced adolescents’ problem behavior. The mediation model found that parent–child conflict and self-control play a direct mediating role between academic pressure and adolescents’ problem behavior. In addition, this study also found that parent–child conflict, self-control, and subjective well-being have important chain mediating effects between academic pressure and adolescents’ problem behavior.

### Academic pressure is an important factor affecting adolescent problem behavior

The study proved that academic pressure has a significant influence on adolescents’ problem behavior. That is, the greater the academic pressure, the higher the risk of adolescents’ problem behavior. At present, China’s education model is still exam-oriented education (Hu and West, 2015). Teenagers are constantly facing various examinations in order to enter better schools. In this process, they bear pressure from all sides, including learning tasks, learning expectations, and interpersonal relationships. Excessive academic pressure is bound to lead to emotional changes in teenagers, which will easily lead to mental illness such as depression in the long run (Anyan and Hjemdal, 2016). Problem behavior is generally the excessive behavior undertaken by teenagers after they bear great psychological pressure. Serious problem behavior includes acts such as suicide and murder (DiCataldo and Everett, 2007), which are not only detrimental to the physical and mental health and social function development of teenagers, but also brings indelible harm to families, schools, and even society. It is particularly important to alleviate the academic pressure on teenagers. To facilitate this, China has adopted a “double reduction” policy. However, at the early stage of the policy’s implementation, there are still many problems. For example, test scores are still an important factor for students to go to higher schools, and the service quality of teachers is inconsistent (Li, 2021). Therefore, it is necessary to continuously strengthen the reform of the education system, take the coordinated development of teenagers’ morality, intelligence, physique, and beauty as guidance, and improve their psychological resilience.

### Parent–child conflict increases the risk of problem behavior in adolescents

The results of the study found that parent–child conflict was an important factor affecting adolescent problem behavior. There was a mediating effect in the association between academic pressure and problem behavior. Family is the primary environment for individual growth, and the influence of family environment on adolescents is very important. Parent–child conflict, as a negative event, can directly affect adolescents’ emotional behavior and increase the risk of problem behavior (Bradford et al., 2007). Academic pressure mainly comes from school, family, and peers, while adolescents’ dependence on family makes them bear more pressure from family. Increasing numbers of parent–child conflicts are rooted in children’s academic performance. Such conflicts will also increase adolescents’ psychological trauma and increase the risk of problem behavior (Rex et al., 1986). Adolescents are a special group whose physical and mental development is not sufficiently sound. A good family environment is particularly important for the formation of their values. Therefore, parents should aim for the healthy growth of their children, attach importance to family relationships, reduce parent–child conflicts, and create a harmonious family ambience.

### Self-control can effectively alleviate adolescent problem behaviors

The study also proved that self-control can directly affect the problem behavior of adolescents and, as a mediating factor, it can alleviate the effect of academic pressure on their problem behavior. One study found that the stronger the self-control ability, the higher an individual’s ability to manage stress and regulate emotions, and the lower the probability of negative behavior to a certain extent (Glenn and Cunningham, 2000; Finning et al., 2017). At the same time, self-control also has a moderating effect, including reducing external environmental factors such as family conflict and poor academic performance (Perrone et al., 2004). Having good self-control can improve adolescents’ academic performance, reduce negative emotions, and resolve academic pressure, thereby reducing the probability of occurrences of problem behavior (Yu, 2010). Therefore, it is necessary to focus on improving the self-control and stress-management ability of young people, channeling negative emotions, and providing better potential conditions for the development of young people’s mental health and social function.

### Improving adolescent subjective well-being can effectively prevent problem behavior

According to the textual content analysis, we found that subjective well-being can effectively alleviate adolescent problem behavior. Subjective well-being, as an important indicator to measure the psychological characteristics of adolescents, is affected by factors such as interpersonal relationships, social support, and social trust. Our study also confirms this view (Renshaw, 2016). The area of adolescents’ activity is mainly between school and home. Family relationships, teacher–student relationships, and classmate relationships constitute adolescents’ interpersonal networks. Negative interpersonal networks will reduce life satisfaction and subjective well-being, and eventually lead to problem behavior (Bücker et al., 2018). This study also partially confirmed this conclusion. Therefore, by strengthening social support and combining family and school, the subjective well-being of adolescents can be jointly improved to reduce the occurrence of bad behavior.

### The complex chain mediating effect of parent–child conflict, self-control, and subjective well-being

Finally, it is worth mentioning that the results of that parent–child conflict, self-control, and subjective well-being play significant chain mediating roles between academic pressure and adolescents’ problem behavior, mainly following three paths. First, academic pressure increases the risk of parent–child conflict, thus reducing subjective happiness, and then increases the risk of adolescents’ problem behavior. Second, academic pressure can reduce self-control, thereby reducing the subjective well-being of individuals and increasing the probability of adolescents’ problem behavior. Third, academic pressure leads to the decline of self-control ability, increases the probability of parent–child conflict, and reduces the subjective well-being of teenagers, leading to problem behavior. Self-control and parent–child conflict affect subjective well-being to a certain extent, thereby affecting adolescents’ problem behaviors. Therefore, there is a need to focus on how to improve the subjective well-being of teenagers. For example, schools can offer psychological courses, strengthen the psychological education of teenagers, and promote or cultivate their character strengths. At the same time, mental education could be integrated into daily teaching, actively guiding the healthy development of young people’s minds and shaping their strong personality traits. In addition, the family relationship is an important part of teenagers’ happiness, so parents should pay more attention to this while educating their children. When parents have a good relationship with their children, they should control their emotions, reduce the frequency of parent–child conflicts, and create a harmonious and happy family relationship.

## Limitations

Some limitations to this study warrant consideration. First, academic pressure, self-control, parent–child conflict, subjective well-being, and problem behavior are cross-sectional in the study and can be further validated using longitudinal data in the future. Second, since the information was gathered from the participants in the study, self-report/recall bias may have existed. However, it is not easy to achieve continued participation among cohorts of adolescents in a cohort study, and the sample size should not be ignored. As a result, our findings with regard to acceptable goodness-of-fit indices deserve to be given more attention.

## Conclusion

The problem behavior of teenagers should be given continuous attention. Academic pressure has a direct positive impact on adolescents’ problem behavior, and parent–child conflict and self-control have a direct mediating effect between academic pressure and adolescents’ problem behavior. In addition, parent–child conflict, self-control, and subjective well-being have important chain mediation effects between academic pressure and adolescents’ problem behavior. The results of this study emphasize that it is necessary to start from the four levels of individual, family, school and society, and combine the external environment and internal performance to strengthen the psychological characteristics of adolescents and reduce problem behavior. For example, schools can offer psychological courses, strengthen the psychological education of teenagers, and promote or cultivate their character strengths. Parents should pay attention to controlling their emotions when they interact with their children, reduce the frequency of parent–child conflicts, and create a harmonious and happy family relationship. Teenagers should also strengthen their cultivation of self-control and reduce problem behavior. In addition, these measures can increase social support and enhance the subjective well-being of adolescents.

## Data availability statement

The original contributions presented in the study are included in the article/supplementary material, further inquiries can be directed to the corresponding author.

## Ethics statement

The studies involving human participants were reviewed and approved. This study was approved by the Ethical Review Committee of Peking University Biomedical (IRB00001052-14010), and all participants signed informed consent. Written informed consent to participate in this study was provided by the participants’ legal guardian/next of kin.

## Author contributions

M-mJ and KG designed the study, analyzed results, drafted, revised the manuscript, and acquisition of funding. M-mJ and Z-yW drafted and revised the manuscript. KG and P-pG analyzed results, and revised the manuscript. All authors contributed to the article and approved the submitted version.

## Funding

This work was supported by the National Natural Science Foundation of China (grant numbers 72074187). The sponsors of the project had no role in the study design, data collection, data analysis, data interpretation, and writing the manuscript.

## Conflict of interest

The authors declare that the research was conducted in the absence of any commercial or financial relationships that could be construed as a potential conflict of interest.

## Publisher’s note

All claims expressed in this article are solely those of the authors and do not necessarily represent those of their affiliated organizations, or those of the publisher, the editors and the reviewers. Any product that may be evaluated in this article, or claim that may be made by its manufacturer, is not guaranteed or endorsed by the publisher.

## Abbreviations

AP: Academic pressure
SC: Self-control
PCC: Parent–child conflict
SWB: Subjective well-being
PB: Problem behavior
CFA: Confirmatory factor analysis
SD: Standard deviation
SEM: Structural equation modeling
